# St. Mark's Hospital

**Published:** 1902-05-03

**Authors:** 


### ST. MARK'S HOSPITAL.

### FESTIVAL DINNER.

As is only fitting in the case of a City charity, the Corporation was well represented at the Festival Dinner at the Hôtel Métropole on the 24th instant to celebrate the 66th anniversary of St. Mark's Hospital. The Lord Mayor (Sir Joseph Dimsdale, M.P.) presided, wearing his jewelled badge of office, and he was supported by his Sheriffs (Mr. Alderman Bell and Mr. Horace Brooks Marshall), who also displayed their civic decorations; while a notable touch of colour was lent to the scene by the gold-laced liveries of the row of resplendent retainers ranged behind the Lord Mayor's chair. Among the guests, in addition to Lord Dungarvan, were Sir Homewood Crawford, the City Solicitor, and Mr. Alderman Pound. Mr. R. Biddulph Martin, M.P., the treasurer, sat next the chairman, and close by were Sir Richard Powell, Mr. Alfred Cooper, and Mr. Sandeman. Altogether the company numbered about 100.

The customary loyal toasts were duly honoured, after which the chairman rose to propose "Success to St. Mark's Hospital." They were met, he said, not only to enjoy a social evening; they had in their minds objects on a higher plane. He was there to plead for a very old City charity. For 66 years St. Mark's Hospital had been doing good work in the City of London, and it was therefore very meet and right that the chief magistrate of the City should occupy the chair at its festival dinner. The hospital was founded in Aldersgate in 1835. It commenced its work in a small and unostentatious manner. It was not long, however, before it became clear that it was necessary to extend the scope of its operations, and it was therefore moved to Charterhouse Square. But this again soon proved inadequate, and in 1851 the hospital was moved to its present situation. Then in 1896 it became necessary to reorganise the whole of the arrangements, with an outlay of £30,000, and a consequent depletion of the capital funds by £25,000. This was a very severe incubus, and had caused those who were responsible for the administration of the charity the greatest anxiety. Then as to the work done. Many of the greatest institutions in the country began in a very small way, and this was no exception. In 1835 the in-patients and out-patients combined numbered only 131. In 1902 the figures had risen to 485 in-patients and 1,314 new out-patients. He thought therefore he might congratulate the institution on having accomplished a most useful and beneficent work. Public attention being just now drawn to the subject, he might mention that of these 35 were cancer cases. That in itself was sufficient to enlist their sympathies. But St. Mark's undoubtedly laboured under certain disadvantages. Whatever they might think as to whether it ought to be so, the fact remained that one could not plead for the class of diseases assisted by special hospitals such as that as one could for hospitals of a more general character. Therefore it was the more incumbent on them all in a quiet but effective manner to interest all their friends and acquaintances in that most useful charity. It was obvious that its work could not be carried on unless the wherewithal was provided by the public. The accounts were not satisfactory—not from an administrative point of view, for that was all that could be desired, but by reason of the small amount given by the public. The annual subscriptions amounted only to £452—a really insignificant sum. The donations were £1,000 last year, but even that was small. The whole reliable income amounted to about £2,000, while it was absolutely essential for the satisfactory administration of the hospital that it should be brought up to £4,500. The actual expenditure last year was £4,300. He was not only appealing to that sympathetic audience, but to the great public, to whom one had never yet appealed in vain, for a hospital which had done good solid work of a special character for the long period of 67 years, and he felt that if he could let the public know that they were absolutely in want, even on their present lines, without considering the extra work they could do if they had the means, he was sure he should not appeal in vain. In asking them to drink to St. Mark's Hospital it gave him the greatest pleasure to join with the toast the name of his old friend and fellow-banker, Richard Biddulph Martin. They had in him a treasurer of an absolutely ideal character. The City of London had known him and his family for generations past as reputable bankers of the highest integrity, and they knew that anything to which he put his hand would be well done. He gave them "Success to St. Mark's and the health of its treasurer."

### "Efficiency before Economy."

The toast was drunk with the greatest enthusiasm, and Mr. Martin, in acknowledging it, deprecated the references to himself but fully endorsed all that the Lord Mayor had said about the hospital. He did not want to enter into the controversy as to the comparative benefits to the community of special or general hospitals, but of this he was sure, that St. Mark's was, in fact, of the very highest service. They laboured under the disadvantage of being determined to treat their cases well. That, in the peculiar cases treated, took time and cost money, and, in consequence, the cost per head largely exceeded that of some other hospitals. But they preferred to be blamed for expensiveness rather than for inefficiency. Their surgeons were known for their eminence throughout Europe. They knew what was requisite for their patients, and the administration preferred to have a rather higher ratio of expense than to turn out patients before they were thoroughly cured so as to increase the number dealt with during the year. If they did things well it must cost money. He had been one of the lay inspectors of the Metropolitan hospitals under the Prince of Wales's, now King Edward's, Fund, and from his experience in that capacity he could assure them that there was none better organised, equipped, or administered than St. Mark's. The public, however, would only give freely to hospitals in debt. As a banker that principle did not commend itself to him, but as treasurer of a hospital he had to recognise it. In conclusion, he emphasised the fact that what he was then pressing for was not large individual amounts, but for a large number of small amounts.

The next toast was the health of the Lord Mayor and Corporation. In acknowledging this, Sir Joseph Dimsdale accepted and endorsed the eulogy passed by the proposer on the Corporation for its benevolence and good works, but concluded by claiming that "palmam qui merui ferat," and therefore he felt their thanks were also due to Mr. Penman, their secretary, to whose energy and singlemindedness in the hard everyday detail work of administration much of their success was due.

The secretary then read the list of subscriptions, which totalled £2,000, and took the opportunity of acknowledging the compliment paid him.

The health of the medical staff was proposed by Mr. Alderman and Sheriff Bell, and suitably acknowledged by Mr. D. H. Goodsall and in a lengthy speech by Mr. F. S. Edwards.

Viscount Dungarvan replied to the toast of the visitors, after which the proceedings, which had been enlivened throughout by music supplied by Carl Heubert's Viennese Band, came to an end.

